# Supplementary material for: Learning curve and surgical outcome of robotic assisted colorectal surgery with ERAS program
Source: Sci Rep. 2022 Nov 29;12:20566. doi: 10.1038/s41598-022-24665-w (PMC9709162; doi:10.1038/s41598-022-24665-w)
Supplement: Supplementary file 2 — Supplementary Information 2. [file 41598_2022_24665_MOESM2_ESM.zip › RAL Raw data-Ñ[▒K/093/HS_ñw╝╨░O▒Kñσ.pdf]

正式摘要(電子簽章：000000219627074)

W52 / W52 ☐英文標題(注意:無英文姓名-請補病人基本檔!) ☐【讀取】憑證

臺中榮民總醫院

出院病歷摘要

轉入號 : 轉出號 :

[redacted]

(8)流水編號	
(9)入院日期	110/06/25 (2021) 直腸外科 W52 - 112
(10)轉科日期	
(11)出院日期	110/06/29 (2021) 住院天數計 4 日

診斷

- 入院 :COLON CA , 20CM FAV
- 出院 :COLON CA , 20CM FAV s/p robotic assitsted anterior resection on 2021/06/26

主訴

- occult blood test positive in health care

病史

- This 57 y/o female with a history of Myoma s/p ATH in 2008 found occult blood test positive in health care went to CRS OPD for help where colonsopy showed S-colon ca. As a result, she was admitted for robotic assisted anterior resection.

體檢發現

-
  - General appearance :
    - easy looking , conscious clear , without cardiopulmonary distress
    - Vital signs自動帶入date 體溫(BT) 脈搏(P) 呼吸(R) 血壓(BP) 身高(BH) 體重(BW) BMI
      20210625 37.5℃ 71次/分 18次/分 142/98mmHg 165.9cm 64.3kg 23.4
    - Vital signs: B.P. 142/98 mmHg , P.R. 71 /min , R.R. 18 /min , B.T. 37.5 ℃
  - HEENT :
    - Eyes: no anemic conjunctiva, no icteric sclera, normal EOM
    - ENT: no otorrhea, no rhinorrhea, no erythematous mucosa, no injected tonsil, no deformity
  - Neck: supple, freely movable, trachea midline,no deformity, no engorged jugular vein , no palpable thyroid or lymph node enlargement
  - Chest:
    - Inspection: Grossly normal, symmetric and fully expansion, no accessory muscle used, no operation scar, no spider angiomata, no gynecomastia
    - Palpation: no palpable mass or nodule, no subcutaneous emphysema, no tenderness
    - Ausculation: clear breathing sound
  - Heart:
    - Percussion: normal heart size
    - Ausculation: regular heart beat, normal S1, S2, no gallop, no murmur
  - Abdomen:
    - Inspection: in
      - flat contour, symetrically, no scar, no stria, no dilated veins, no rashes
    - Ausculation: normoactive bowel sound, no abdominal bruits
    - palpation: soft, no muscle guarding or rigidity, no local tenderness, no rebounding pain, no palpable mass, no palpable pulsation
    - Percussion: no shifting dulllness, no hepatomegaly, no splenomagaly
  -

Peripheral pulsation :	CA	BA	RA	FA	PA	PTA	DPA
Right side :	++	++	++	++	++	++	++/+++
Left side :	++	++	++	++	++	++	++/+++

- - Genito-anus: no tenderness or palpable mass lesion or rectal shelf, normal external genitalia, normal anal tone, brown stool

CRS CONDITION

-
    - Lung

手術日期及方法

- 手術日期及方法20210626 Robot Assisted Colectomy(L)

住院治療經過

- After admission, pre op sruvey was OK an doperation was done smoothly. SHe tolerated the rpocedure well and could stand oral feeding gradually. Unde relatively stable conditions, she will be discharged.

合併症

一般檢查

- 累積報告：

DATE	TIME	ALB	TP	BIL.T	BIL.D	ALKP	AST	ALT	LDH	AMY	LIPASE	NH3	Ferritin	UA
1100625	1547			0.70		72		11	197					
1100626	1344	3.9		0.70	0.17		17	10						

DATE	TIME	WBC	RBC	HGB	HCT	MCV	PLT	BLST	NEUT	BAND	SEG	LYM	MON	EOS	BAS
1100625	1548	4250		12.3	36.6		191		59.8			34.4	4.2	1.4	0.2
1100626	1344	8690		11.6			161		83.6			14.5	1.3	0.5	0.1

DATE	TIME	NA	K	CL	BUN	CREAT	eGFR	CA	Mg	P	CRP	ESR	PCT	D-Dimer	LACTATE
1100625	1547	141	3.9					9.7							
1100626	1344	141	3.6		8	0.82									

DATE	TIME	GLUCOSE	HbA1C	CHOL	TG	HDL	LDL	KETONE	CK	CKMB	TRO-T	PT-P	APTT-P	NT-pro BNP
1100625	1548											10.4	27.0	

特殊檢查

病理報告

其它

出院時情況

- 3.改門診治療

出院指示

-

藥名	劑量	劑量單位	用法	頻次	數量	數量單位	現況	天數	單位
Acetal tab 500mg	1	TAB	PO	Q6HPRN	28	TAB	TH	7	天
(口)Takepron OD tab 30mg	1	TAB	PO	QDAC	7	TAB	TH	7	天
Mag. Oxide tab	2	TAB	PO	TID	42	TAB	TH	7	天
$Biofermin tab	2	TAB	PO	TID	42	TAB	TH	7	天
Through tab	2	TAB	PO	QN	14	TAB	TH	7	天

出院計劃

主治醫師 2062F 蔣鋒帆 / 住院醫師 2309E 古朋育

正式摘要(電子簽章：000000219627074)

W52 / W52

doctorRank:B
